# Silicon etch with chromium ions generated by a filtered or non-filtered cathodic arc discharge

**DOI:** 10.1080/14686996.2016.1140308

**Published:** 2016-02-23

**Authors:** Daniele Scopece, Max Döbeli, Daniele Passerone, Xavier Maeder, Antonia Neels, Beno Widrig, Alex Dommann, Ulrich Müller, Jürgen Ramm

**Affiliations:** ^a^ nanotech@surfaces laboratory, Empa, Swiss Federal Laboratories for Materials Science and Technology, Überlandstrasse 129, CH-8600, Dübendorf, Switzerland; ^b^ Ion Beam Physics, ETH Zürich, Otto-Stern-Weg 5, CH-8093, Zürich, Switzerland; ^c^ Oerlikon Surface Solutions AG, Oerlikon Balzers, Iramali 18, 9469Balzers, Liechtenstein; ^d^ Laboratory for Nanoscale Materials Science, Empa, Swiss Federal Laboratories for Materials Science and Technology, Überlandstrasse 129, CH-8600Dübendorf, Switzerland; ^e^ Center for X-ray Analytics, Empa, Swiss Federal Laboratories for Materials Science and Technology, Überlandstrasse 129, CH-8600Dübendorf, Switzerland

**Keywords:** Adhesion, metal ion etch, filtered arc, nucleation, surface binding energy, TRIDYN, 40 Optical, magnetic and electronic device materials, 402 Multi-scale simulation, 212 Surface and interfaces, 305 Plasma/Laser processing, 503 TEM, STEM, SEM, 504 X-ray/Neutron diffraction and scattering

## Abstract

The pre-treatment of substrate surfaces prior to deposition is important for the adhesion of physical vapour deposition coatings. This work investigates Si surfaces after the bombardment by energetic Cr ions which are created in cathodic arc discharges. The effect of the pre-treatment is analysed by X-ray diffraction, Rutherford backscattering spectroscopy, scanning electron microscopy and in-depth X-ray photoemission spectroscopy and compared for Cr vapour produced from a filtered and non-filtered cathodic arc discharge. Cr coverage as a function of ion energy was also predicted by TRIDYN Monte Carlo calculations. Discrepancies between measured and simulated values in the transition regime between layer growth and surface removal can be explained by the chemical reactions between Cr ions and the Si substrate or between the substrate surface and the residual gases. Simulations help to find optimum and more stable parameters for specific film and substrate combinations faster than trial-and-error procedure.

## Graphical abstract

**Figure:**
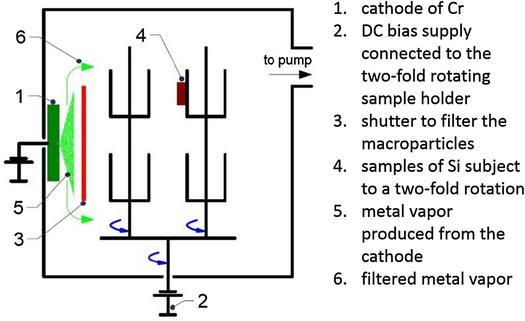

## 1. Introduction

In physical vapour deposition (PVD) the bombardment of substrate surfaces with ions before deposition is an established process to improve the coating adhesion. For conductive substrates, the bombarding energy of the positively charged ions can be controlled by a negative bias applied to the substrates. A surface treatment with inert gas ions such as argon supports the desorption of gaseous species and removes surface contaminants by sputtering. The situation is different if, instead of inert gases, ionized metal vapour is utilized. Depending on the magnitude of the applied bias, the treatment can result in layer growth or surface removal by sputtering. The removal of surface atoms by metal ions is known as metal ion etch (MIE) [1,ch.8.5,^2,3,4^]. This denomination does not usually distinguish if only metal ions were utilized or if argon was added to increase the efficiency of sputtering. Generally, MIE is used to remove surface atoms.

In industrial deposition technology, the ionized metal vapour for the substrate pre-treatment is frequently generated by cathodic arc sources. The arc discharge produces from the cathode material (target) highly ionized metal vapour with different charge states and neutrals [1]. In addition, the arc discharge generates macroparticles, so called droplets. These droplets are undesirable for some applications, because they may reduce the mechanical and thermal stability of the coatings. The droplets also influence the result of the MIE because they carry no charge, are different in size and contribute to layer growth, i.e. reduce the efficiency of sputtering. For the pre-treatment of the substrates with ions, the substrates can either be exposed directly to an arc source or, if droplets have to be avoided, the vapour from the arc source can be filtered. The first case will be named here *non*-*filtered MIE* (NF-MIE) and the second *filtered MIE* (F-MIE).

If substrate surfaces are bombarded with pure ionized metal vapour (ions, neutral vapour, macroparticles), there is a range of low bias voltages for which layer growth can be observed, while at higher (more negative) bias the substrate surface is removed. The transition bias range between layer growth and surface removal depends on the arc discharge (filtered, non-filtered), the composition of the ionized vapour, the residual gas components and pressure, and the substrate material. Better understanding of the basic physical and chemical processes at the substrate surface governing material deposition and removal will support a faster process development in industrial PVD coating applications.

The present work investigates this transition range in a PVD production system for Si substrates bombarded with Cr ions. The results of the pre-treatment are investigated for NF-MIE and F-MIE configurations as a function of substrate bias. The experimental data are compared with TRIDYN simulations [5–7]. These simulations are not able to describe the full complexity of the experiments but are extremely useful for a discussion of trends and a more detailed understanding of the transition region.

## 2. Experimental details

### 2.1. Sample preparation

**Figure 1.**
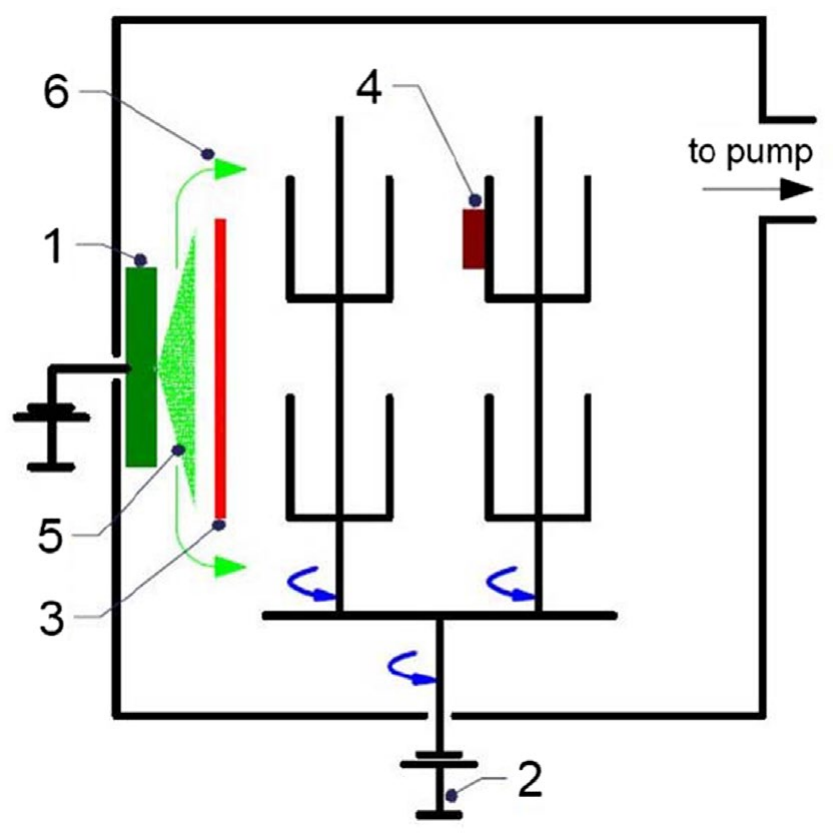
Schematics of the experimental set-up for the MIE in the deposition system. The picture shows: the cathode of Cr (1), the DC bias supply connected to the twofold rotating sample holder (2), the shutter to filter the macroparticles (3), the samples of Si subject to a twofold rotation (4), the metal vapour produced from the cathode (5) and the filtered metal vapour (6). The shutter (3) is fixed during F-MIE and removed for NF-MIE.

The samples are prepared in an INNOVA batch-type production system of Oerlikon Surface Solutions AG (Figure 1). The deposition chamber is evacuated to a pressure of 2 × 10^−4^ mbar before the cathodic arc source, which produces the ionized metallic vapour for the substrate pre-treatment, is ignited. These vacuum conditions are typical for the deposition of temperature-sensitive components requiring temperatures below 300°C. This is also the reason why it is resigned to heat the vacuum chamber to accelerate desorption of gases and water from the inner chamber walls. Elemental Cr targets (cathodes) are utilized to produce the ionized metallic vapour with a DC discharge current of 140 A, resulting in a Cr evaporation rate of 12.2 g h^–1^ in both NF-MIE and F-MIE. The cathodes are installed at the inner chamber wall as schematically shown in Figure 1.

In the case of NF-MIE the substrates are directly exposed to the arc source. For the filtered case (F-MIE), there are different implementation approaches described in the literature [1,ch.7.4]. In our experiments, a fixed water-cooled shutter is installed in front of the cathode, to protect the substrate surfaces from the deposition with metallic droplets and from the condensation of neutral metallic vapour. This is a simple design which can be realized in every deposition system equipped with arc sources. The fixed shutter does not, however, completely block the ion current. Bias voltages between –50 V and –1200 V are applied to the substrates in order to modify the bombardment energy of the Cr ions. No additional gases (such as Ar) are added to the chamber, i.e. the arc discharge is self-sustaining and produces Cr vapour only. Although there are no additional gases fed to the vacuum chamber, the plasma created by the arc discharge current initiates gas desorption, especially of water vapour, from the inner chamber walls and fixtures. These gases are dissociated and activated in the arc discharge. Simultaneously, the metallic vapour “pumps” these gases efficiently due to gettering which is manifested in the drop of total chamber pressure right after arc ignition. The substrates are mounted on standard substrate holders and experience a twofold rotation during ion bombardment (Figure 1). The total substrate ion current (I_Bias_, Table 1) measured for the substrate including substrate holder in F-MIE is between 0.7 and 0.8 A, while it amounts to about 6 A for NF-MIE. The substrates are immersed in the plasma of the arc discharge. They charge to about –5 to –10 V against ground if they are left at floating potential, indicating that a plasma sheath is formed. The Si substrates are wet-chemically cleaned before loading to the deposition system. For each bias value, the Si substrates are bombarded for 30 min with the ionized metal vapour. At the end of this treatment, their temperature does not exceed 100°C for all bias voltages in F-MIE, but can exceed 500°C for direct exposure for NF-MIE.

**Table 1.** Process parameters of the NF-MIE and F-MIE experiments.

NF-MIE	**U**_**Bias**_**[-V]**	**I**_**Arc**_**[A]**	**I**_**Bias**_**[A]**	**Cr [10**^**16**^**at/cm**^**2**^**]**
	50	140	5	300
	200	140	5.3	135
	400	140	5.5	35
	600	140	6	5.7
	800	140	6.5	21
F-MIE				
	50	140	0.7	20.0
	200	140	0.7	13.6
	400	140	0.7	3.3
	600	140	0.8	11.6
	800	140	0.8	9.1
	1000	140	0.8	4.1
	1200	140	0.8	3.3

A summary of the experimental conditions is given in Table 1.

### 2.2. Coating characterization

For a qualitative comparison of NF-MIE with the F-MIE treatments, the surface and fracture cross-sections of the coatings are investigated in a LEO 1530 scanning electron microscope (SEM). In this work we use a cross-section scanning electron microscopy and will define it as X-SEM.

In addition, the samples are characterized by Rutherford backscattering spectrometry (RBS) [8] at the ETH Laboratory for Ion Beam Physics. Measurements are performed using a 2 MeV ^4^He beam under normal incidence to the sample surface with a beam spot size of 1 mm. Backscattered particles are detected at an angle of 168° towards the incident beam direction by a silicon PIN diode detector with an energy resolution of 12 keV (FWHM). The collected RBS data are fitted by simulations using the RUMP software [9]. For the thinnest deposits the resolution of the RBS measurement is not sufficient to resolve the exact depth profile of the material composition and the degree of implantation or sub-plantation of the layer. Therefore only the net areal density in number of metal atoms per Å^2^ is determined and this is the quantity compared with our simulations with TRIDYN. In the case of NF-MIE some layers are thick enough for RBS to reveal resolved compositional depth profiles, which will be discussed in more detail below.

In order to check possible crystal phases created during deposition, the samples are analysed with X-ray diffractometry (XRD) on a PANalytical X’Pert PRO MRD instrument located at Empa Thun (CH) manufacturer: PANalytical using Cu-Kα radiation in a 2θ mode with 1° grazing incidence angle. This small angle is used to get maximum information of the surface of the coating and to avoid the signal from the substrate. The crystallography open database (COD) is used to identify the crystallographic phases [10,11].

The F-MIE samples are characterized with X-ray photoemission spectroscopy (XPS) in depth analysis. Depth profiles are acquired on a Physical Electronics Inc Chanhassen Instrument located in Empa Dubendorf (CH) [12] using monochromatized Al Kα radiation (hν = 1486.7 eV) and a hemispherical capacitor electron-energy analyser equipped with a channel plate and a position-sensitive detector. The electron take-off angle is 45° and the beam diameter used is around 150 μm. The analyser is operated in the constant pass energy mode at 58.7 eV and for the spectra a step size of 0.25 eV is used for all measurements. The binding energy is calibrated using Cu 2p_3/2_, Ag 3d_5/2_ and Au 4f_7/2_ at 932.62 eV, 368.21 eV and 83.96 eV, respectively to within ± 0.1 eV [13]. To compensate for possible surface charging, built-in electron and argon ion neutralizers are used. The base pressure of the system was below 5 × 10^−7^ Pa. Sputtering was performed with Ar^+^-ions with 1 kV acceleration voltage and scanned over an area of 2 × 2 mm which results in a typical sputter rate on Ta_2_O_5_ of 2 nm min^–1^. The real sputter rate on chromium, chromium oxide or silicon may differ from the actual one by up to a factor of 2; however, the direct comparability is certainly better than 10%. The Cr 2p, Si 2p, O 1s and C 1s peaks were measured. Depth profiles are analysed using the software MultiPak 8.2B [12] and peak areas are determined after a Shirley background subtraction. The atomic concentrations are calculated using the corrected relative sensitivity factors as given by the manufacturer and normalized to 100 at.%.

### 2.3. TRIDYN simulations

In this work we adopt the Monte Carlo code TRIDYN (version HZDR) [5–7], used successfully in previous works by other authors [14,15]. In a previous investigation [16], our attempts to simulate the results of F-MIE were not satisfactory and this is the reason to investigate this topic in greater detail in this work. This code uses a binary collision approximation to model the multiple and cascade collisions between incoming ions and the atoms of the substrate. We intentionally neglect non-kinetic effects such as diffusion or chemical reactions [17,18] and focus on the redistribution of energy and atoms via collisions. The parameters used in the simulations in both cases are reported in Table 2, justified in section 3 and briefly discussed here.

**Table 2.** Parameter utilized for TRIDYN simulations. The beam compositions for NF-MIE are the ones actually used with the neutrals included.

Parameter	Non-filtered MIE	Filtered MIE
Beam composition	Cr^4+^ (0.6%), Cr^3+^ (12.60%), Cr^2+^ (40.8%), Cr^1+^ (6.0%), Cr^0^ (40.0%), from [1]	Cr^1+^ (100%)
Final fluence [atoms/Å^2^]	300	20
Number of histories	200,000	20,000
SBV (Cr,Si) [eV]	6.69 (CrSi_2_)	6.69 (CrSi_2_)
SBV (Cr,Si) [eV] test to interpolate the outliers	X	6.69 (CrSi_2_)
SBV (Cr,O) [eV] Test to interpolate the outliers	X	10.20 (Cr_2_O_3_)
SBV (Cr,Cr) [eV]	4.12	
SBV (Si,Si) [eV]	4.70	

The total final fluence is extracted from the RBS measurements for the bias of –50 V for which sputtering has little influence and sticking probability is assumed to be 1. The kinetic energy of the ions is determined by the bias voltage applied in experiments and by the charge of the ions imposed in the simulations with no further broadening considered. The surface binding energy (SBE) of each atomic species is calculated from the local material composition via the binding energy matrix SBV describing mutual binding energies between all types of atoms present. The diagonal SBV components (Cr–Cr and Si–Si) are adopted from the TRIDYN code [10]. The off-diagonal SBV component is computed from the formation enthalpy of different compounds (Table 2) observed in our XRD results discussed below (section 4) and other values for comparison in selected cases. It has been checked that the number of simulated particle histories in the Monte Carlo calculation is large enough to exclude any significant statistical variation.

The TRIDYN simulations executed for NF-MIE (Figure 6(a)) are based on the ion charge state distribution given in Table 2, based on the closest conditions reported in the literature [1,p.508]. In addition to that, it is assumed that about 40 at.% of the Cr projectiles are neutrals, without a specific classification between droplets and atoms. This assumption is justified by the comparison of the evaporation rate of Cr (12 g h^–1^) with the total substrate current at the substrate holder (about 6 A) and assuming that some of the neutral Cr will not be deposited at the substrate surface but at the chamber walls. The simulations of F-MIE are performed by utilizing a pure Cr^1+^ beam. However, slight modifications of this assumption do not alter the trend considerably.

Based on these parameter sets, the areal density of Cr as a function of substrate bias, the surface recession (i.e. etching or deposition depth) as a function of Cr fluence for different substrate biases, and the fraction of Cr as a function of depth for different substrate biases are calculated and utilized for the comparison with experimental results.

## 4. Results and discussion

**Figure 2.**
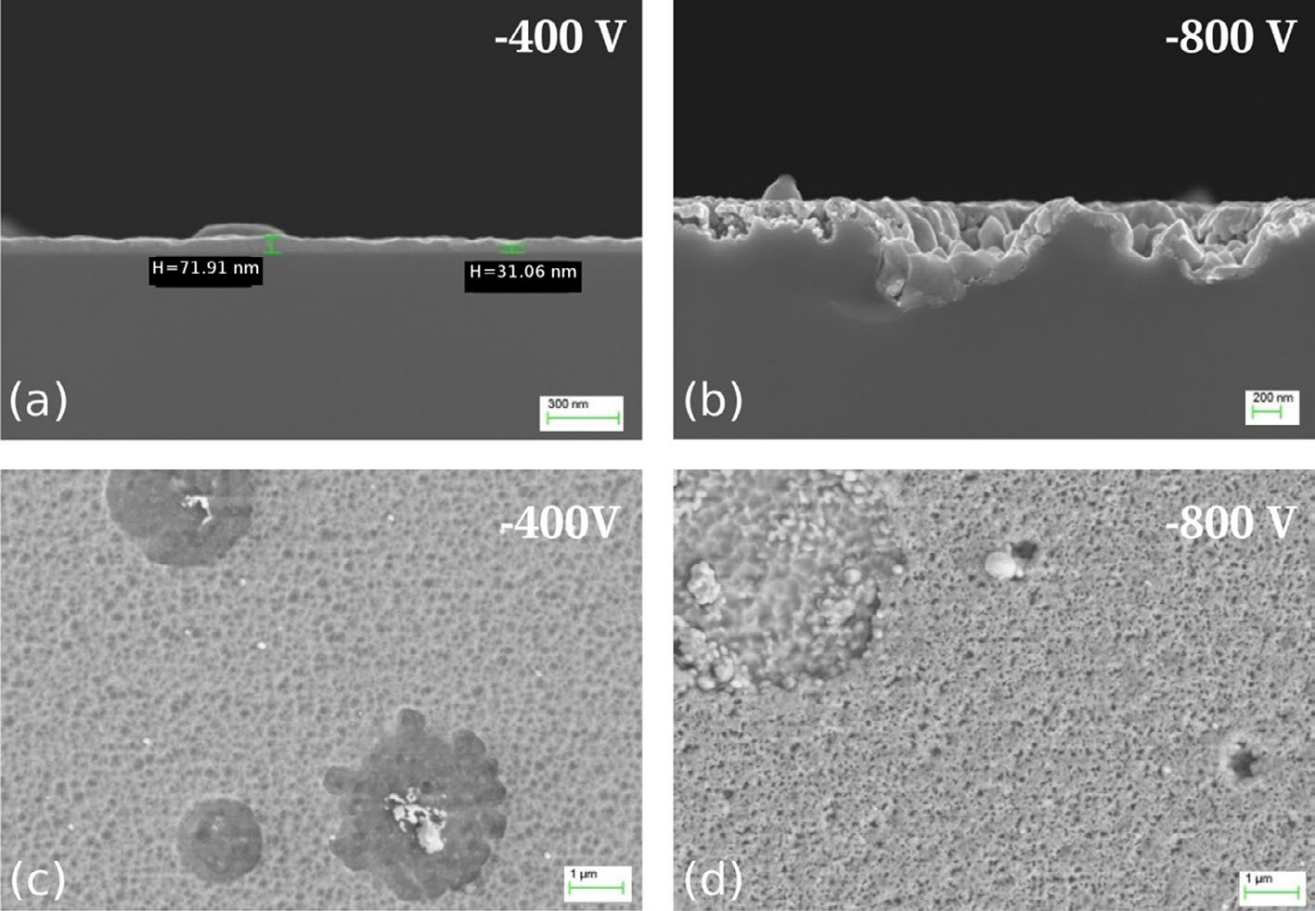
SEM micrographs of the Si samples obtained in NF-MIE. (a) and (b) show the cross section of the samples for –400 V bias voltage and –800 V bias, respectively. For both bias voltages, the surface appearance is shown in (c) and (d), respectively. See discussion in section 4.

Figure 2(a) and (b) shows the cross section of substrates treated in NF-MIE for substrate bias of –400 V and –800 V. At –400 V (Figure 2(a)), a coating can be detected. However, the determination of the thickness is difficult because the layer is not uniform in thickness. Values between 30 nm and 70 nm can be estimated. No coating is visible at the substrate which was biased with –800 V (Figure 2(b)). Instead, the substrate surface is damaged to a depth of 500 nm and more. The top view of the sample surface obtained at –400 V (Figure 2(c)) shows a fissured coating including circular areas with droplet residues. The appearance of these areas suggests that the droplets were removed during the NF-MIE process. The structure of the surface for –800 V (Figure 2(d)) shows a similar porosity, however, with more pronounced etching effect for the droplet regions. These regions do not contain Cr residues anymore. The findings indicate the difficulties to determine the end of layer growth in the NF-MIE process and the detrimental influence of droplets not only for the coating uniformity but also for a defined transition to etching. Given the occurrence of the damaged substrate surface at –800 V, no investigations of higher bias voltages were performed.

**Figure 3.**
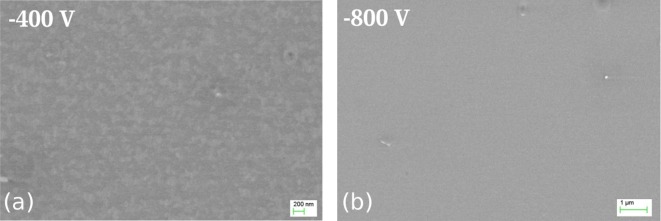
SEM micrographs of the Si surfaces obtained after F-MIE for (a) –400 V and (b) –800 V. The surface does not show crater formation like it does for samples of NF-MIE.

The substrate ion current is one order of magnitude lower for F-MIE, resulting in a coating thickness which cannot be determined by X-SEM investigations. The top view of the substrate surface shows a non-continuous layer with weak indications of island growth for –400 V bias (Figure 3(a)). For –800 V (Figure 3(b)) the surface is featureless, with the exception of few defects ascribed to particles. The transition between layer growth and etching is difficult to determine in the case of NF-MIE because of droplet induced etching effects. In F-MIE, it is difficult as well because a thickness measurement is not possible by standard methods like X-SEM. For thin film development, however, it would be of interest to determine the parameters for which the substrate material first appears at the surface and may influence the layer nucleation and the chemistry of the film-substrate interface.

**Figure 4.**
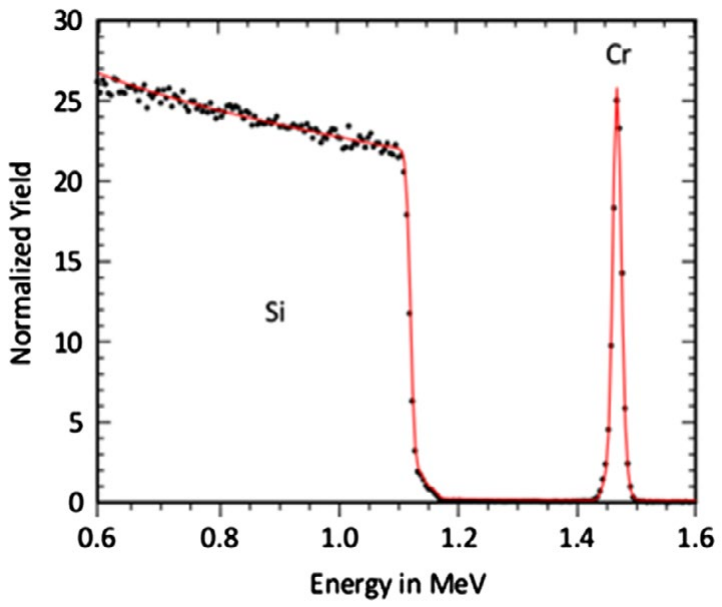
Typical RBS spectrum for F-MIE sample (–800 V bias).

Therefore, it was investigated if RBS could provide a better quantification of the transition region between growth and etching. In Figure 4, a typical RBS spectrum is given. The spectrum was measured for the sample which was exposed to F-MIE at –800 V bias. All spectra of the samples prepared in F-MIE show a Cr signal which is well separated from the Si substrate. This allows an accurate estimation of the total Cr areal density. The depth resolution of the technique, however, does not permit a distinction if the Cr layer is above the Si surface (layer growth) or intermixed with the substrate, which is also the case for the oxygen which may be present in the spectra.

**Figure 5.**
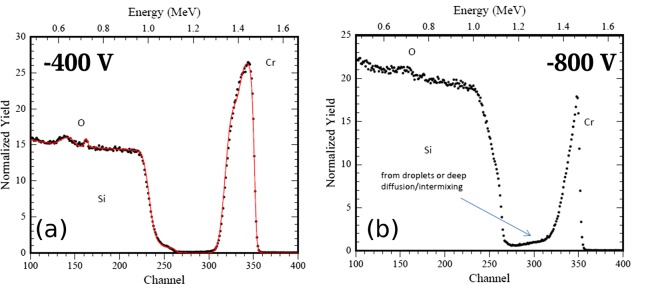
RBS spectra obtained from NF-MIE. For the bias of –400 V (a), the spectrum indicates oxygen accumulation at the interface and at the surface and additional Si intermixing in the interface. For the bias of –800 V (b), enhanced intermixing of Si and Cr is observed. The Cr low energy tail is either caused by droplets on the surface or Cr diffusion into the substrate.

All RBS spectra obtained from the samples of NF-MIE between –50 V and –600 V bias have also well separated Cr signals. An example is shown in Figure 5(a) for the experiment with –400 V bias. This sample was tilted during analysis by 57° to increase depth resolution. The spectrum indicates a thin oxide at the surface of the sample and an oxide in the interface. In addition, the comparison with RUMP simulations suggests a Si profile in the coating with a Si/Cr ratio of about 0.15 at the interface and zero at the surface, indicating that a Si–Cr intermixed region is formed before the Cr layer growth. For the bias of –800 V (Figure 5(b), sample not tilted), the Cr peak has a distinctive tail towards lower energies. This indicates Cr diffusion into the Si substrates or can result from increased surface roughness e.g. by droplets (see Figure 2(b)). The spectrum also shows in the Si signal at the surface a lower Si value than at greater depth, which might be explained by silicide or oxide formation. For all NF-MIE samples, oxide contaminations are detected in the interface. This indicates that at least in the beginning of the MIE the residual gas, mainly water vapour, influences the result of the MIE.

**Figure 6.**
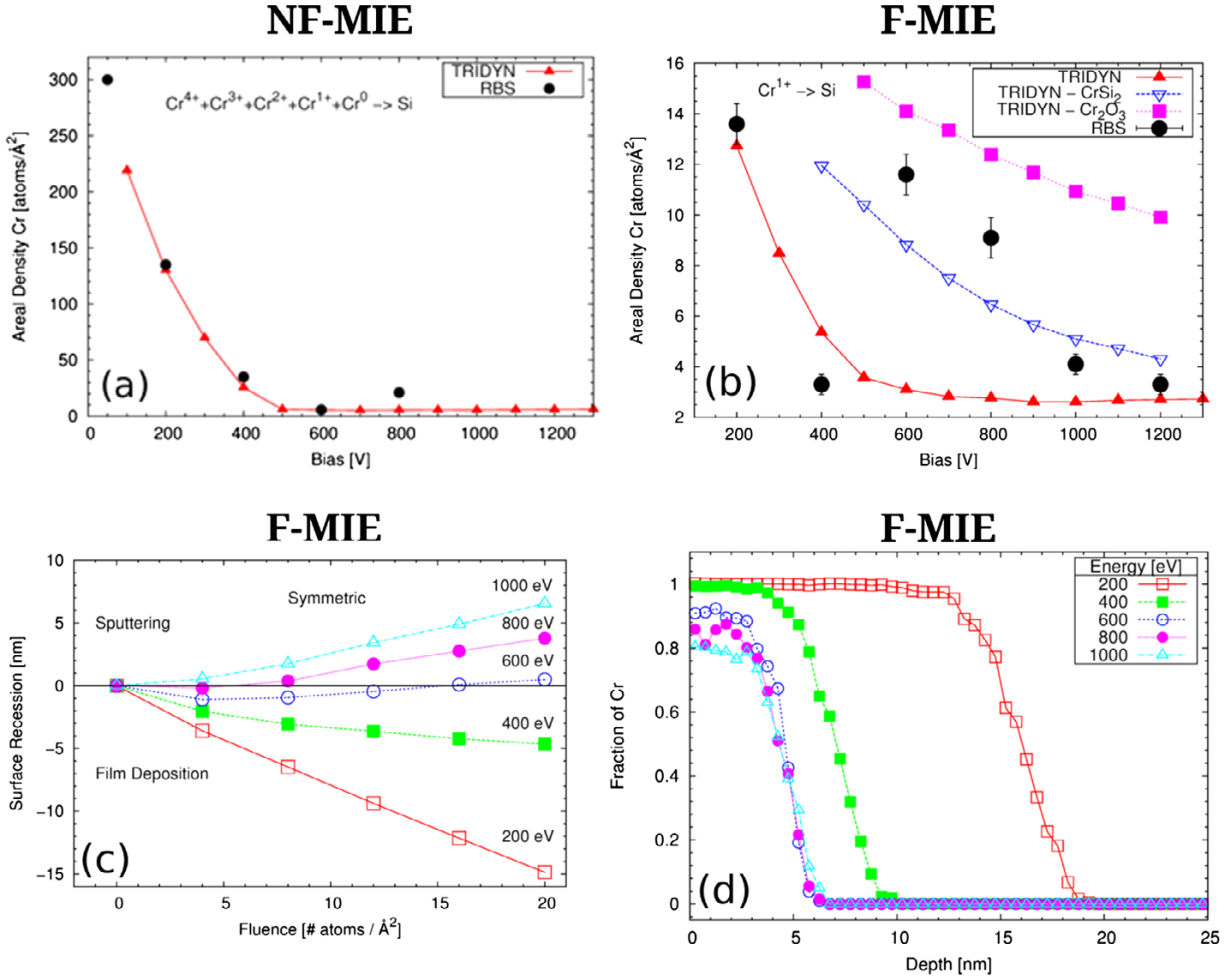
TRIDYN simulations (red curves) with the parameters specified in Table 2 superimposed on the RBS experimental data (black points) for NF-MIE (a) and F-MIE (b), respectively. In (b) the blue and purple curves are obtained with modified values of the SBE for the Cr silicide and the Cr oxide reported in the key, representing the limit of the possible mixed phases created (see text for discussion). (c) Surface recession in F-MIE for the different substrate bias (see section 4 for discussion). (d) Cr concentration profile versus depth for different substrate bias for F-MIE at the final fluence of 20 atoms/Å^2^.

The measured Cr areal density (black dots) as a function of the substrate bias is given in Figure 6 for NF-MIE (a) and F-MIE (b) experiments. If, for sake of simplicity, we consider ionized vapour and kinetic processes only, some general trends should be reflected in these curves [19]. For energies up to a few hundred eV, sputtering is negligible and the bombarding atoms can penetrate only a few nm into the material. Disregarding chemical effects and volatility of certain elements and compounds, a film grows with virtually the same elemental composition as the impinging beam with an intermixed interface layer between substrate and film. Depending on the flux, the time span between intermixed interface formation and complete layer formation is different, i.e. the exposure time of the sample surface to vacuum ambient is different. For higher energies, layer growth turns into layer removal and the erosion of surface atoms starts due to sputtering. Simultaneously, the ion implantation depth increases with bombarding energy and most of the substrate atoms are removed at the beginning of the bombardment. After a layer with a thickness corresponding to the implantation range has been removed, more and more previously implanted ions are also sputtered and finally an equilibrium state is established.

To confirm these general trends for our experiments, TRIDYN simulations are performed with the parameters discussed in section 2.3 and reported in Table 2. The parameters of our simulations were chosen to obtain a reasonable agreement for the regime of layer growth at the lowest bias and are not based on measurements, as was discussed above. It was not the intention to completely describe NF-MIE and F-MIE under our conditions with TRIDYN, but to become able to better separate effects purely induced by energetic atomic collisions from those surface modifications that cannot be described by the binary collision approximation (on which TRIDYN is based) alone, such as diffusion, chemical processes and phase formation. This set of simulations is thought to extend and complement the one reported in [16] to take into account the two possible explanations highlighted above as suggested from experiments: oxides or silicides. In [16] we investigated the presence of oxygen in the beam due to the water vapour in the chamber. In this work we consider the option of no oxygen with formation of silicides or oxides in the compounds (but not in the beam explicitly), as discussed in detail below.

The results from TRIDYN simulations are given in Figure 6(a) and (b) (red line) for NF-MIE and F-MIE, respectively. With the chosen parameters, the simulations are brought in fair agreement with the experiments for layer growth.

Figure 6(c) reports the trend of the surface recession in function of substrate bias and for different fluences. Negative values of this quantity correspond to deposition (growth of coating), and positive values to sputtering (recession of the coating). For small fluences, it indicates dynamical equilibrium conditions at –800 V, shifting to –600 V for higher fluences. In Figure 6(d), the distribution of the Cr versus depth at the final fluence of 20 atoms/Å^2^ for different substrate bias is shown. According to these simulations, Si will appear at the substrate surface at –600 V bias and may contribute to reactions. This is also the range for which the RBS data disagree with TRIDYN simulations (Figure 6(b)).

Indeed, based on our discussion (and from TRIDYN), we would expect a monotonic decrease of the atomic density with increased bias. On the contrary, an increase in areal densities is measured for NF-MIE at –800 V and for bias voltages of –600 V and higher for F-MIE.

**Figure 7.**
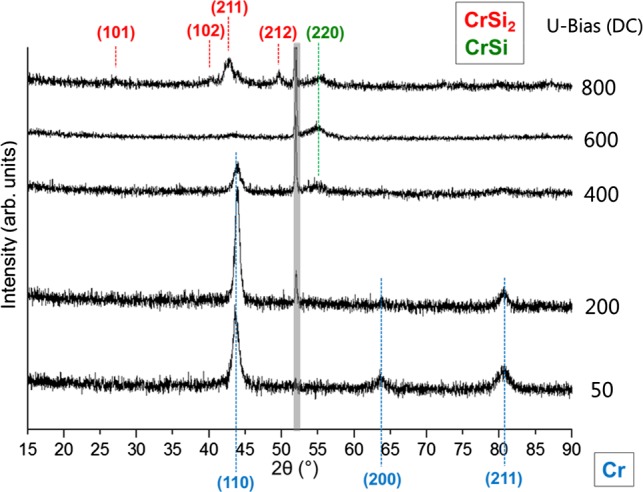
Grazing incidence XRD spectra of the substrate surfaces treated with NF-MIE for different substrate bias. This is a 2θ measurement with a grazing angle of 1°.

This discontinuity in the curves can be understood as a jump in surface binding energy (by using the “TRIDYN” jargon) indicating chemical reactions at the surface, as outlined below for the case of F-MIE. To understand the reason behind the unexpected increase of the Cr areal densities, XRD grazing incidence measurements are performed to explore the phase formation close to the surface of the substrates obtained in NF-MIE (Figure 7). The analysis shows only the Cr Bragg peaks in the bias range between –50 V and –400 V which is in accordance with the net layer growth also observed in SEM analysis. At –400 V an additional peak appears which can be attributed to CrSi (COD 1010034) [20] and at –800 V CrSi_2_ (COD 1010033) [20] is formed at the substrate surface. The XRD analysis supports the idea that for small Si concentrations at the substrate surface CrSi is formed and for higher Si concentrations (–800 V) enough Si is present at the surface to form CrSi_2_. However, it is likely that in NF-MIE also thermal effects play a role. This means that chemical reactions occur during the bombardment of the Si surface with Cr vapour produced by the non-filtered arc source. In the spectrum, no oxide phases can be detected, despite the fact that oxygen is found in the RBS spectra. This indicates that possible formed oxides are amorphous.

The layers formed during F-MIE are too thin for XRD analysis. The performed XRD grazing incidence analysis (not shown here) could not identify a silicide. However, assuming a detection limit of about 3.5 nm, a silicide formation of a few monolayers cannot be excluded.

**Figure 8.**
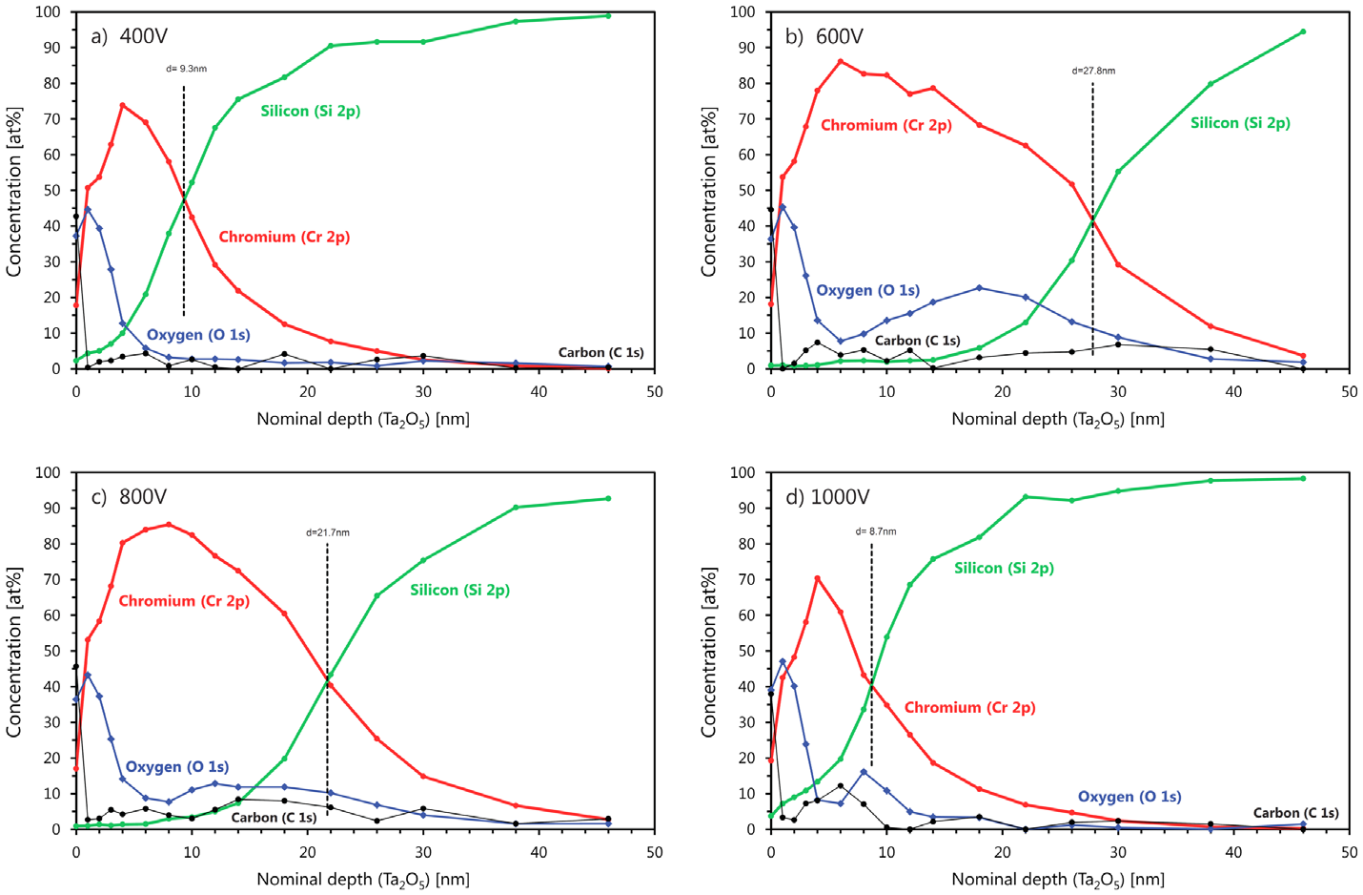
XPS measurements on the F-MIE samples. From panel (a) to (d): 400, 600, 800, and 1000 V bias. The depth is not calibrated. The amount of oxygen in the outliers 600 and 800 V is considerably higher than the other two: this supports the idea of formation of oxides against the one on silicides. See text for discussion.

To find the reason for the increase of the Cr areal density measured by RBS and the depth profile of oxygen, XPS was performed for these samples. The results are shown in Figure 8 for bias voltages between –400 V and –1000 V. In all XPS spectra, an oxygen surface peak is visible. The peaks have similar shapes and thickness. The shoulders in all Cr signals are correlated with the oxygen, indicating a surface oxidation of Cr. This surface oxide is most probably due to the exposure of the sample to ambient after the deposition process and therefore not related to F-MIE. At –400 V (Figure 8(a)) and –1000 V (Figure 8(d)) substrate bias, a small amount of Si can also be detected at the substrate surface, indicating that not only chromium oxide is formed. The thickness for the Cr containing regions in the two substrate surfaces is about the same. However, only at –1000 V do we see a clear indication of oxygen in the interface region to the silicon substrate. For –600 V (Figure 8(b)) and –800 V (Figure 8(c)), the Cr depth profile shows a larger thickness with a Cr concentration of about 70 at.%. In addition, a higher oxygen concentration between 10 and 20 at.% was measured, indicating that an oxide was formed. These results indicate partial oxidation of the sample surface during F-MIE, especially in this bias range. A qualitative explanation for this is not straightforward. However, the comparison with TRIDYN simulations can be of help in this case as well.

We can conclude that the higher areal densities obtained in the transition between layer growth and surface removal can be simulated with higher surface binding energies. If we assume silicide or oxide formation and therefore (partially) replace the surface binding energy (SBV) of the Si–Si, Cr–Cr or Si–Cr by the SBV values of silicides or oxides we can easily reduce the sputter yield and reproduce the higher areal densities. This is performed in Figure 6(b) for the –600 V and –800 V values for the CrSi_2_ and Cr_2_O_3_ compounds. In this case the higher SBV and SBE values generate a higher amount of Cr in the sample and a better agreement with the points at 600 and 800 V bias. The two curves in Figure 6(b) (blue and purple) represent the extreme cases of a complete coverage of the surface by pure silicide or pure oxide (TRIDYN allows only the simulation of complete coverage and not mixed phases). The idea of the simultaneous formation of both Cr silicides and Cr oxides (both likely under-stoichiometric), as suggested by our XPS data, implies that the corresponding amount of Cr in this mixed phase lies between the two curves depicted in Figure 6(b), thus in fair agreement with the experimental RBS data.

TRIDYN is not only able to provide a good estimation for the substrate bias at which zero growth equilibrium has to be expected (i.e. the moment when chemical reactions are more likely to take place). It can also reveal details in the temporal evolution of the surface composition which may well be a possible cause for the suspected enhanced oxide formation for F-MIE. If a value for the particle flux is given, the curves of surface position versus fluence in Figure 6(c) can be transformed into surface position versus absolute time. The curves for 600 and 800 V bias are of special interest here. They demonstrate that even for a bias with net surface removal there is always an initial deposition of Cr due to implantation. The total time span of this transition phase shown in the figure corresponds to 30 min. Thus, surface atoms have a considerable amount of time to interact with the environment and create compounds that are more resistant to sputtering.

## 4. Conclusions

The transition between layer growth and surface removal for the treatment of Si surfaces with pure metallic Cr vapour from a cathodic arc source is difficult to control in production systems. The direct exposure of the substrates to the ionized vapour and high flux of ions may result in chemical reactions between the ionized metal and the atoms in the substrate surface which can turn in pronounced surface roughening. Silicides are formed already at a substrate bias (–400 V) for which net layer growth takes place. Increased reactivity is observed for the regions where droplets occur, indicating that excess Cr accelerates surface removal. This fact and a rather abrupt transition between layer growth and surface damage restrict the application of this approach and make the stable tuning of NF-MIE for tiny layer thickness impossible.

A better control can be obtained if the ions are produced by a filtered arc source. Such a source does not only generate droplet-free ionized vapour, but also reduces the ion flux and total fluence which in turn allows better control of the transition between growth and surface removal. It also reduces the thermal budget in the MIE process. This was shown for F-MIE with typical ion currents about an order of magnitude lower than corresponding currents in NF-MIE. Under these conditions, the filtered Cr vapour does not damage the Si surface. However, oxide formation is observed in the transition region probably due to longer residence times of atoms at the surface and indicates that the control of the residual gases is essential for a reliable F-MIE process operating at low fluences. An F-MIE can be critical if the ion flux is comparable with the impingement rate of the residual gas. A comparison with TRIDYN helps predict the bias region between layer growth and etching in which the substrate surface is changing slowly and becomes more susceptible to the influence of residual gases, diffusion and chemical reactions with substrate atoms. It is the region for which the atoms of the substrate appear at the surface. At this bias, the surface is not yet covered by growth nor experiences high etch rates and reactions forming compounds with larger SBV are possible. This region is very important to engineer interfaces. The fit of the experimental data in the growth regime with TRIDYN and the agreement or disagreement for the remaining curve indicate whether chemical reactions must be considered. Once F-MIE is described in this way, TRIDYN simulations can be extended to other substrate materials to allow the prediction of zero growth conditions.

## Disclosure statement

No potential conflict of interest was reported by the authors.
